# The Influence of Hemp Extract in Combination with Ginger on the Metabolic Activity of Metastatic Cells and Microorganisms

**DOI:** 10.3390/molecules25214992

**Published:** 2020-10-28

**Authors:** Taja Žitek, Maja Leitgeb, Andrej Golle, Barbara Dariš, Željko Knez, Maša Knez Hrnčič

**Affiliations:** 1 Laboratory for Separation Processes and Product Design, Faculty of Chemistry and Chemical Engineering, University of Maribor, SI-2000 Maribor, Slovenia; taja.zitek@um.si (T.Ž.); maja.leitgeb@um.si (M.L.); zeljko.knez@um.si (Ž.K.); 2 Faculty of Medicine, University of Maribor, Taborska 8, SI-2000 Maribor, Slovenia; barbara.daris@um.si; 3 National Laboratory for Health, Environment and Food, Prvomajska ulica 1, SI-2000 Maribor, Slovenia; andrej.golle@nlzoh.si

**Keywords:** hemp, ginger, antioxidant, total phenol, metastatic melanoma cells WM-266-4, *Staphylococcus aureus*, *Escherichia coli*, *Candida albicans*

## Abstract

This study presents an investigation of the anticancer and antimicrobial ability of a combination of ginger and cannabis extracts in different ratios (1:1, 7:3 and 3:7). Extracts were obtained using various methods (Soxhlet extractions, cold macerations, ultrasonic extractions and supercritical fluid extractions). The antioxidant activity and the presence of total phenols were measured in the extracts, and the effect of the application extracts in various concentrations (c = 50, 20, 10, 5, 1, 0.1, 0.01 mg/mL) on cells was investigated. Higher values of antioxidants were measured at the ratio where ginger was predominant, which is reflected in a higher concentration of total phenols. Depending on the polyphenol content, the extracts were most effective when prepared supercritically and ultrasonically. However, with respect to cell response, the ratio was shown to have no effect on inhibiting cancer cell division. The minimum concentration required to inhibit cancer cell growth was found to be 1 mg/mL. High-performance liquid chromatography (HPLC) analysis also confirmed the effectiveness of ultrasonic and supercritical fluid extraction, as their extracts reached higher cannabinoid contents. In both extractions, the cannabidiol (CBD) content was above 30% and the cannabidiolic acid (CBDA) content was above 45%. In the case of ultrasonic extraction, a higher quantity of cannabigerol (CBG) (5.75 ± 0.18) was detected, and in the case of supercritical fluid extraction, higher cannabichromene (CBC) (5.48 ± 0.13) content was detected, when compared to other extraction methods. The antimicrobial potential of extracts prepared with ultrasonic and supercritical extractions on three microorganisms (*Staphylococcus aureus*, *Escherichia coli* and *Candida albicans*) was checked. Ginger and cannabis extract show better growth inhibition of microorganisms in cannabis-dominated ratios for gram-positive bacterium *S. aureus*, MIC = 9.38 mg/mL, for gram-negative bacterium *E. coli*, MIC > 37.5 mg/mL and for the *C. albicans* fungus MIC = 4.69 mg/mL. This suggests guidelines for further work: a 1: 1 ratio of ginger and hemp will be chosen in a combination with supercritical and ultrasonic extraction.

## 1. Introduction

The incidence of malignant melanoma, an extremely invasive and metastatic type of skin cancer, is increasing [1]. Since chemotherapy is mostly ineffective, advanced malignant melanoma has a poor prognosis [2,3,4]. Consequently, intense research has been performed on the anticancer effect of natural compounds in the treatment of melanoma. Natural products derived from plants have already been recognized to possess therapeutic potential for various diseases, including cancer [5]. Plant alkaloids such as catharanthus alkaloids, colchicine, etoposide and taxol [6,7,8,9,10,11] are used as anti-cancer (“antineoplastic” or “cytotoxic”) chemotherapy drugs.

Hemp is considered as medicinal plants with health promoting effects, comprising anti-inflammatory ability [12,13,14,15,16,17,18,19,20,21,22,23,24]. In the present study, cannabis was used in combination with ginger, which has good antioxidant activity [12,13]. Hemp (*Cannabis sativa* L.) is a dioecious plant of the order Rosales, family *Cannabaceae* and genus *Cannabis* [14]. It grows in various habitats and altitudes, from sea level all the way to the alpine foothills of the Himalayas, from where it probably originates [15]. Hemp consists of three subspecies: *Cannabis sativa*, *Cannabis ruderalis*, and *Cannabis indica* L. [16]. In 2004, with the amendment of the Rules on the conditions for obtaining a permit for the cultivation of hemp (Official Gazette of the Republic of Slovenia, No. 40/11 and No. 36/15), hemp began to appear again on Slovenian arable land. In terms of production between 2015 and 2017, the most widely cultivated varieties were Fedora 17, USO 31, and KC Dora [17,18,19]. Currently, 69 varieties of cannabis are authorized for commercial use by the European Community [20]. According to the literature, cannabis has variability within the same genus. In the pharmaceutical field, two phenotypes are generally considered: “drug-type Cannabis,” which is rich in psychoactive components (such as Δ9-THC), and “fibre-type,” or industrial cannabis, with a high content of non-psychoactive cannabinoids (such as CBD). The use of a variety (*Cannabis sativa*) whose THC content does not exceed 0.2% in the dry matter of the plant is permitted. Our research focused on a non-psychoactive species; Kc Dora, which is grown in Slovenia [21,22,23].

**Figure 1:**
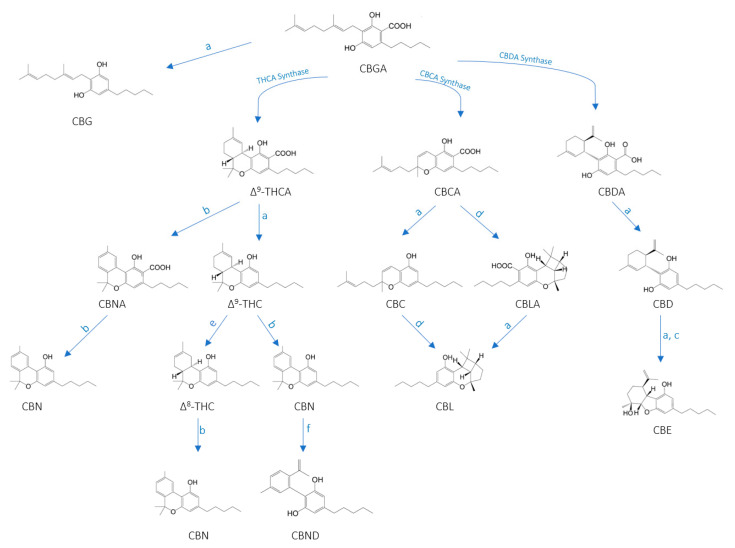
Chemical structures and biosynthesis of the major cannabinoids present in *Cannabis sativa L*: a = heating, b = oxidation, c = photo-oxidation, d = photo-irradiation, e = isomerization, f = photochemical conversion [21,39]**.**

Several chemical compounds have been identified in the *C. sativa* plant, including terpenes, carbohydrates, fatty acids and their esters, amides, amines, phytosterols, phenolic compounds and cannabinoids [17,24,25,26]. The five most common cannabinoids found in cannabis are cannabidiol (CBD), Δ9-tetrahydrocannabinol (THC), cannabichromen (CBC), cannabigerol (CBG) and cannabinol (CBN), whose structure can be seen in Figure 1. All five show strong antibacterial effects against various methicillin-resistant *Staphylococcus aureus* (MRSA). Recent research has demonstrated that plant leaves can be an important source of antimicrobial components [27,28,29]. *Escherichia coli* [27], *Bacillus megaterium* [30], *Staphylococcus aureus* [27,31], *E. coli* (ATCC 25922) [27,28], *Bacillus subtilis* and *Candida albicans* have been found to be sensitive to various plant extracts [27,28,29,30,31,32,33,34,35]. Cannabis extracts also have antioxidant activity. From a pharmaceutical point of view, CBD is the most promising among non-psychoactive cannabinoids, as it has both antioxidant and anti-inflammatory properties. The cannabidiolic acid (CBDA) component has antimicrobial and anti-inflammatory properties, while the CBG component has anti-inflammatory, antimicrobial and analgesic properties [17,23,36,37,38].

Cannabinoids such as THC and CBD are decarboxylation products. Carboxyl groups contain a specific arrangement of carbon and oxygen atoms. Decarboxylation is the process of removing a carboxyl group (a carbon atom double bonded to an oxygen atom) from a molecule (a carbon atom is removed from a carboxylic acid (THCA and CBDA)). In cannabinoids, this reaction occurs when the cannabinoids are heated. A suitable temperature for decarboxylation is around 120 °C [29,31,32,33,34]. Cannabinoids are biosynthesized in acid form in plant tissues; they can then form their decarboxylated relatives under the action of heat and light by spontaneous decarboxylation, as shown in Figure 1 [21,39]. The figure also shows other minor cannabinoids, including cannabichromenic acid (CBCA), cannabichromene (CBC), cannabinolic acid (CBNA) and cannabinol (CBN). It can be seen that the initial form is CBGA, which is converted from one carbon form of cannabidoid to another by heating, oxidation, photo-oxidation, photo-irradiation, isomerization or photochemical conversion. The image shows several components—Cannabigerolic acid (CBGA), cannabigerol (CBG), delta-9-tetrahydrocannabinolic acid (Δ^9^-THCA), cannabichromenic acid (CBCA), cannabidiolic acid (CBDA), cannabinolic acid (CBNA), delta-9-tetrahydrocannabinol (Δ^9^-THC), cannabichromene (CBC), cannabicyclolic acid (CBLA), cannabidiol (CBD), cannabinol (CBN), delta-8-tetrahydrocannabinol (Δ^8^-THC), cannabidinodiol (CBND), cannabicyclolic (CBL) and cannabielsoin (CBE).

Ginger (*Zingiber officinale*) is a member of the plant family that includes cardamom and turmeric. In previous studies [40], we investigated turmeric and demonstrated that it is a strong source of antioxidants and that turmeric extract has a significant effect on the metabolic activity of melanoma cells (WM-266-4), even at the lowest extract concentration of 0.001 mg/mL [36]. According to recent studies, ginger also has antioxidant [12,13,41,42], anti-microbial [43], anti-cancer and anti-inflammatory activity. The antioxidant effect of ginger has been studied using various methods. For example, the CO_2_ extract of ginger has high polyphenol content. It manifests very good scavenging of DPPH and lessens its reducing capacity. The extract can be used as an antioxidant at early stages of fat oxidation. The structures of more than 50 antioxidants isolated from the rhizomes of ginger, where isolated antioxidants were divided into two groups, gingerol related compounds and diarylheptanoids, have been determined. Studies suggest the antioxidant activity might be due not only to the radical scavenging activity of antioxidants but also their affinity to antioxidants with substrates [12,13]. Most antioxidant components exhibit higher activities in alcohol media, as determined by different assays. Hence, apart from its medicinal properties, ginger can also be used as an antioxidant supplement [42]. Several review articles have shown that ginger contains monoterpenoids, sesquiterpenoids, phenolic compounds and their derivatives, aldehydes, ketones, alcohols and esters, which provide a wide antimicrobial spectrum against various microorganisms [43].

The type of solvent and the choice of isolation method strongly influence the yield and content of active compounds. According to the literature, ethanol, methanol or ethyl acetate are most often used as solvents, but some studies used hexane [44], acetone [45,46], dichloromethane [47] and kerosene ether (PE) [48]. Lone et al. found out that the yield of cannabinoids derived using acetone and ethanol was the highest [49]. Ethanol is generally the preferred solvent, as it has a low melting point. Studies report good results using supercritical fluid extraction (SFE) with CO_2_ and ethanol as a co-solvent, where pressures ranged from 80 to 300 bar and temperatures from 20 °C to 70 °C [50,51,52,53,54,55,56]. Most studies show that the optimal pressure is 250 bar at a temperature range from 40 °C to 60 °C to achieve high efficiency and yield of active components [49,50,51].

In our study, we used SFE and the optimal conditions were chosen using the same pressure and temperature ranges as hemp, but the extraction was carried out without co-solvent at 200 bar and 300 bar and at 40 °C and 60 °C. Conventional extractions using ethanol as a solvent (ultrasonic extraction (UE), Soxhlet extraction (SE) and cold maceration (CM) [51,54,55,56]) were also carried out for comparison. In this study, the dried material of both plants was mixed and later extracted. The aim of this research was to explore possible synergistic effects of these mixture of extracts on the melanoma cells and microorganisms. The motive for the choice of these materials was in the high antimicrobial and anticancer potential of hemp and the high antioxidative potential of ginger.

## 2. Results and Discussion

This work presents the yields obtained by the extractions performed (SE, CM, UE and SFE). The results present contents of antioxidants and total phenols and the influence of extracts on the inhibition of division or growth of melanoma cells WM-266-4 and microorganisms (*Staphylococcus aureus*, *Escherichia coli* and *Candida albicans*).

Table 1 presents the obtained yields of the extractions. The extractions were repeated twice and the mean values are presented. SFE with CO_2_ gave the highest efficiencies at 300 bar and 60 °C. The yields of conventional extractions did not differ significantly from the SFE yields; only the UE yield stands out slightly. The highest yield was calculated for x11 extract (η = 9.12%). However, the results of extract x_73_ and x_37_ are slightly higher than other values. According to the results, the material ratio has no significant effect on yields. Most of the studies reviewed report a 20 to 40% yield on supercritical cannabis extraction [50,51,52,53,55,56] but they all used ethanol as a co-solvent in SFE. In this work, supercritical CO_2_ extraction was performed without co-solvents. In the case of SFE of ginger, the yield results are around 5% [57,58]. A similar yield was obtained by UE of ginger [59]. For further research, we could try to add ethanol or try the compression process, as did Aladić et al., or to use a process of oil extraction from Cannabis sativa L. seeds performed by cold pressing, followed by extraction with supercritical CO_2_ (60 °C, frequency of 20 Hz and nozzle of ID 6 mm) [60]. The pulse regime shows better performance than traditional co-solvents at a constant concentration in the solvent stream, and it achieves the same extraction efficiency with lower solvent consumption and much shorter extraction times [61].

Table 2 presents the calculated values of antioxidant activity given in percentages and the values of total phenols given in mg GAE/100 g of material. The x_73_ extract, in which ginger material predominated, had a visibly higher antioxidant content, regardless of the extraction methods applied. This was expected, as other studies report high antioxidant levels in ginger (A = 96%) [62]. Very low concentrations of c = 2 mg/mL were required to achieve 50% inhibitory efficacy, according to the literature [63]. In sample x_37_, in which ginger is present in the lowest proportion, antioxidant activity is lowest (A = 30–40%). The highest antioxidant activity (A = 79.872%) was achieved by SFE at 300 bar and 60 °C. Porto et al. came to the same conclusions [54]. The highest content of total phenols was achieved with UE in the x_11_ extract, 773,008 mg GAE/100 g material (Table 2). However, in the x_73_ extract, high TP values were obtained for most extractions (Table 2). Compared to the available results of conventional extractions in the literature, UE prevailed in high results of bioactive components. TP was high using UE for all extracts. Agarwal et al. investigated the interaction of UE power time and solvent dilution, using methanol as a solvent [64]. Methanol gives similar results to ethanol [65]. According to our statistical analysis, the optimal time to reach the maximum content of TP is 15 min, power 90 W; the maximum concentration of methanol is 80%. Peak TP values were measured at 313 mg GAE/100 mg [64]. In our study, twice these values were measured. Smeriglio et al. achieved TP = 267.5 with cold-pressed extract from seeds of industrial hemp (*Cannabis sativa* L.), results comparable to ours from CM [66].

**Figure 2:**
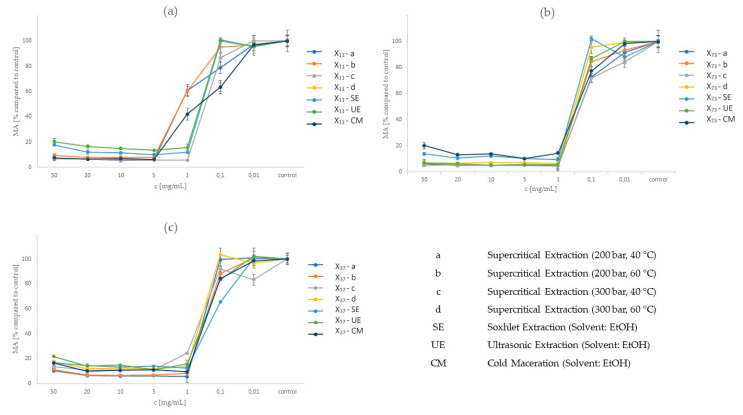
Metabolic activity at different concentrations of ginger and industrial hemp mixture using different extractions (SCF-a, SCF-b, SCF-c, SCF-d, SE, UE, CM). (**a**) x_11_ extract prepared in a 1:1 ratio of ginger to hemp. (**b**) x_73_ extract prepared in a 7:3 ratio of ginger to hemp. (**c**) x_37_ extract prepared in a 3:7 ratio of ginger to hemp.

Ginger-industrial hemp extract was efficient in inhibiting the metabolic activity of WM-266-4 cells at concentrations ranging from 50 mg/mL to 1 mg/mL. The metabolic activity of the cells decreased to about 15% (Figure 2). Figure 2 shows three graphs for each mixture, where graph (a) represents metabolic activities when the materials (ginger: hemp) are in a ratio of 1:1; graph (b) represents metabolic activities when the materials are in a ratio of 7:3; and graph (c) represents metabolic activities when the materials are in a ratio of 3:7. The *x*-axis shows the concentrations of extracts that were applied to the cells. The *y*-axis shows the metabolic activity of the cancer cell relative to the control after the extract was added. All extracts achieved a significant change in the metabolic activity of the melanoma cells at an extract concentration of 1 mg mL. The exception was in the case of x_11_ extract (SFE, 200 bar, 60 °C), x_11_ (SE) and x_11_ (CM). This achieved a significant change in the metabolic activity of the melanoma cell at a higher extract concentration of 5 mg/mL. The prepared mixture has not previously been applied to these cells. These are the first studies on the response of metastatic skin cancer cells (WM-266-4) to the selected extracts.

**Figure 3:**
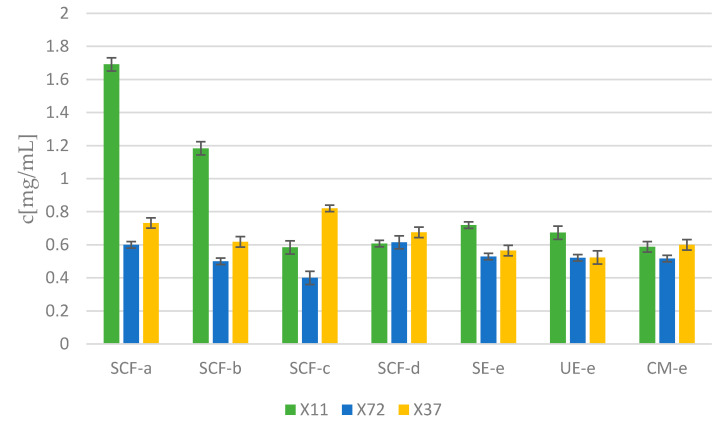
Concentration at which the metabolic activity of a melanoma cell is equal to 50% (IC50).

Figure 3 shows IC50 values, which means the concentration at which the metabolic activity of a melanoma cell is equal to 50%. This concentration generally ranges between 0.4 and 0.8 mg/mL.

**Figure 4:**
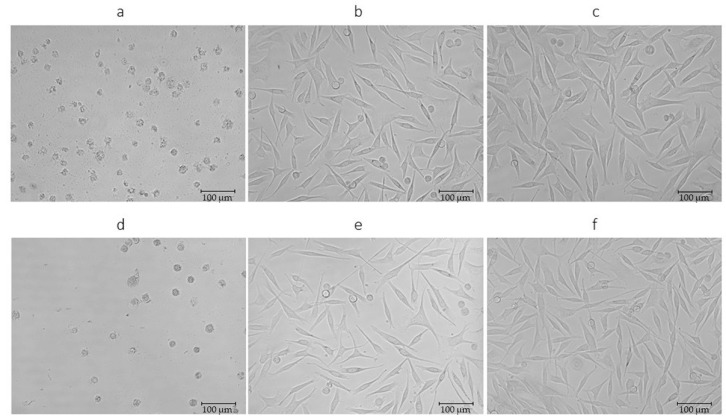
Morphology of WM-266-4 cells exposed to extract x_37_. (**a**) The response of melanoma cells upon application of 1 mg/mL concentration of SFE extract at 300 bar and 60 °C. (**b**) The response of melanoma cells upon application of 0.1 mg/mL concentration of SFE extract at 300 bar and 60 °C. (**d**) The response of melanoma cells upon application of 1 mg/mL concentration of UE extract. (**e**) The response of melanoma cells upon application of 1 mg/mL concentration of UE extract. (**c**,**f**) represent the control. Magnification 200×.

Figure 4 shows a change between the concentration of 1 mg/mL, where there is a significant decrease in the metabolic activity of the melanoma cell, and the concentration of 0.1 mg/mL, where the figure is quite similar to the control. In Figure 4, the first line presents images of a melanoma cell to which an x_37_ extract was applied (obtained by SFE at a temperature of 60 °C and 300 bar). The second line represents melanoma cells after application of extract x_37_ (obtained by UE). A jump is clearly visible between Figure 4a,b,d,e. At a concentration of 1 mg/mL (Figure 4d), no cell divisions are visible, and the melanoma cells have lost their original shape, as shown in Figure 4c,f.

**Figure 5:**
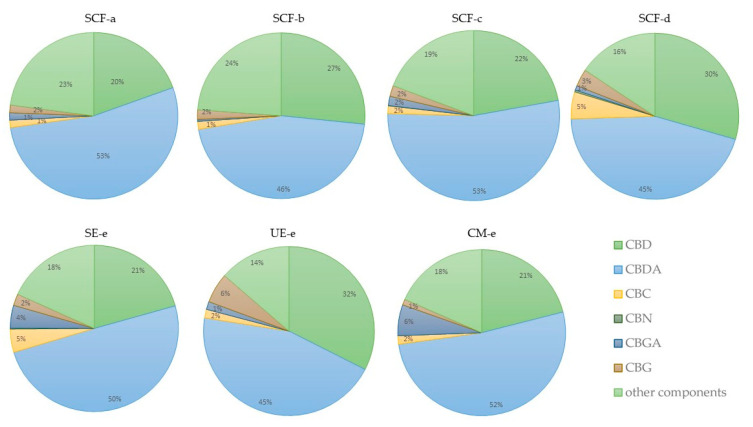
Cannabinoid component of *Cannabis Sativa* L. extract. Analyses were performed in duplicate and values expressed as cannabinoid percentage. The procedure for obtaining the percentage results is described in Section 3.2.5.

The diagrams in Figure 5 show the proportions of the contents of the selected components in the analysed extract (x_11_). The measurement uncertainty is <2% for CBD and CBDA components and < 0.2 for all other specified components (Table S1). The diagrams demonstrate that the CBDA component predominates in all extracts; the content of CBDA is approximately twice as high as the content of CBD. Such results were expected, as the material was not decarboxylated. The content of the components in the extracts did not differ significantly. However, SCF-d extract and UE extract had slightly higher CBD content (32%). Also, UE extract had a higher content of CBG (6%) than other extracts, and SCF-d higher content of CBC (5%). CBD is the foundation of our research, but CBC and CBG are also very important components, as they have antifungal, anti-inflammatory, analgesic and antibiotic actions, important properties for this research [67].

**Figure 6:**
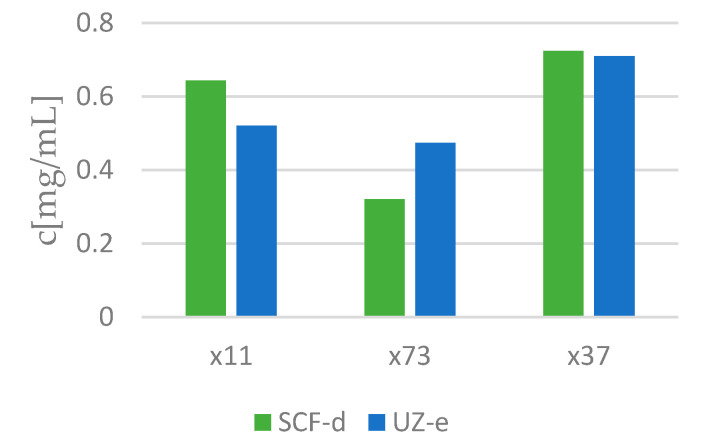
The graph represents EC50, the concentrations at which 50% of the antioxidant activity of the selected extract was measured.

Based on antioxidant activity, the content of total phenols and HPLC analysis, the following extracts were selected for further work: extracts obtained using UE (x_11_ UE-e, x_73_ UE-e and x_37_ UE-e) and SFE extracts at 300 bar and 40 °C (x_11_ SFE-d, x_73_ SFE-d and x_37_ SFE-d) were selected and then applied to several microorganisms: *S. aureus*, *E. coli* and *C. albicans*. The results are presented in Table 3, which shows the minimum inhibitory concentration (MIC). Figure 6 presents the EC50 values of these extracts. The graph shows that in extracts in which ginger (x_73_) predominates, a lower concentration is required to achieve 50% antioxidant activity.

Gram- positive bacteria have a thicker peptidoglycan layer than gram-negative bacteria, but they are more receptive to certain cell wall targeting antibiotics because of the absence of the outer membrane. Table 3 shows that for the gram-positive organism S. aureus, a lower concentration (MIC) is required than for the gram-negative organism *E. coli.* Many Cannabis sativa L. extracts in different solvents (ethanol [60,66], methanol [68,69,70], hexane [69,70], petroleum ether [70], aqueous [71], acetone [31], etc.) and different extraction processes have been studied and applied to bacteria or fungi. In our study, ethanol (for UE) and CO_2_ (for SFE) were used as solvents.

In S. aureus, the marginal concentration MIC values were immediately apparent, while in *E. coli* we could not confirm the MIC concentration at all. Kaur et al. came to similar conclusions when they achieved a MIC of 6.25 mg/mL for ethanolic extract [65]. In our study, a MIC value of 4.69 mg/mL for x_37_ extract was achieved. In the case of the gram-negative organism *E. coli*, however, it has been reported that a much higher concentration (MIC > 50 mg/mL) is required, which is also our conclusion. Therefore, among our extracts, the UE extract (x_73_), in which industrial hemp is predominant (70%), had the best effect on the bacterium S. aureus. Wasim et al. also found that the MIC of ethanol extract was approximately 5 mg/mL for gram-positive S. aureus, which is comparable to the antibiotic cephalexin [72,73].

Fungus C. albicans showed the same MIC value, 4.69 mg/mL, for all extracts, with the difference that the extracts obtained with SFE began to show a change in colour at the lowest concentration of 0.07 mg/mL. In the research of Nissen et al., the MIC value was 2 mg/mL with industrial hemp oil extract [29]. Studies using nystatin as a reference standard have shown that the measured MIC value of hemp extract for the fungus C. albicans is about 1 mg/mL, which is slightly lower than the concentration determined in our study [73]. According to our results, the ratio of ginger to industrial hemp had no effect in inhibiting the action of the fungus C. albicans. The antimicrobial results described in this study (which used a combination of ginger and hemp) are close to the results of studies using singly ethanolic hemp extract [31,59,73,74,75,76,77]. Antibiotics otherwise have slightly lower MIC values; for example, Frassinetti et al. found positive control in growth in the presence of the standard antibiotics gentamicin and vancomycin. The gram-negative microorganism *E. coli* ATCC 25922 showed a MIC value of 1 mg/mL. The same MIC value was found for the gram-positive bacterium S. aureus [77]. Panpatil et al. studied the effect of ginger on the growth of test organisms and reported, for S. aureus, MIC = 125 mg/mL and for *E. coli* MIC = 175 mg/mL [59], which are even higher values than in this study. Onyeagba et al. performed antimicrobial studies of ginger and a combination of ginger and garlic. The antimicrobial trimethoprime-sulfamethoxazole (primpex) was used as a sensitivity control. Ginger and garlic, as individual ethanol extracts, showed no in vitro growth inhibition of S. aureus and C. albicans, but, in combination, they inhibited S. aureus [76]. Lee et al. found that a SFE extract of ginger was an efficient inhibitor of the growth of S. aureus and *E. coli* [75].

Farha et al. investigated individual cannabinoids and determined MIC values for cannabinoid analogues against MRSA USA300. They demonstrated that the mechanism of action of the cannabigerol proceeds by targeting the cytoplasmic membrane of gram-positive bacteria and demonstrates the in vivo efficacy of cannabigerol in a model of muscle systemic infection caused by *S. aureus*. Cannabinoids have also been shown to be effective against gram-negative organisms whose outer membrane is permeable, where cannabigerol acts on the inner membrane [74].

## 3. Materials and Methods

### 3.1. Materials

Ginger, roughly ground, was purchased from Alfred Galke GmbH (Samtgemeinde Bad Grund, Germany) and industrial hemp (leaf and bud) was purchased from local growers in Slovenia. The type of hemp was Kc Dora, which was air dried at 38 °C. Kc Dora is characterized by a vegetative cycle of 145 days and grows up to 250 cm.

The chemicals used were 2,2-diphenyl-1-picrylhydrazyl (DPPH) (Sigma Aldrich, Darmstadt, Germany, ≥97.0%), carbon dioxide (Messer; MG-Ruše, Slovenia, purity 2.5), chloric acid (HCl), (Sigma Aldrich, 37%), dimethyl sulfoxide (DMSO), ethanol (EtOH), (Sigma-Aldrich, HPLC grade, ≥99.9%), ferrous sulfate heptahydrate (Fe(SO_4_) × 7 H_2_O) (Sigma Aldrich), Folin-Ciocalten reagent (FC), (Sigma Aldrich), gallic acid (Sigma Aldrich, 97.5–102.5%), methanol (MeOH), (Honeywell, Charlotte, NC, USA, LC-MS CHROMASOLV^®^, ≥99.9%), *n*-butanol (Sigma Aldrich, ≥99.5%) and sodium(V)carbonate (Na_2_CO_3_), (Sigma Aldrich, ≥99.9%).

### 3.2. Methods

#### 3.2.1. Preparation of Material for the Extraction Process

Dried ginger and industrial hemp were ground to a powder, with a water content of 10 wt%. Three different combinations of ground materials were prepared. The first combination (x_11_) had a ratio of 1:1 (ginger:industrial hemp), the second (x_73_) 7:3, and the third 3:7 (x_37_). Subsequently, various extractions (SE, UE, CM, SFE) were used to prepare the extract. The final extract was stored in a container and kept in a freezer at 4 °C for further analysis.

#### 3.2.2. Extractions

Conventional extractions (SE, UE, CM) were performed with ethanol solvent. The solvent for the SFE was CO_2_. Ethanol and CO_2_ were chosen because the former isolates mainly polar components and the latter non-polar components. Co-solvent was not added to supercritical CO_2_; otherwise, it could increase its solvent power in favour of polar molecules and enhance the cannabinoids’ extraction efficiency; greater solvent power could mean lower process selectivity [78].

In the Soxhlet extraction (SE), 20 g of mixed material (x_11_, x_73_, x_37_) was ground in a mixer and extracted with 200 mL of ethanol at 70 °C. Cold maceration (CM) was performed at a mixing speed of 300 rpm with the same amount of solvent and material. The mixture for ultrasonic extraction (UE) (40 kHz) was of the same concentration as the other two conventional extractions but at 25 °C. After 3 h of extraction, the ethanol was removed at 40 °C under reduced pressure with a rotary evaporator (Büchi Rotavapor R-114, Flawil, Switzerland). The extracts were weighed, and the yield calculated.

**Figure 7:**
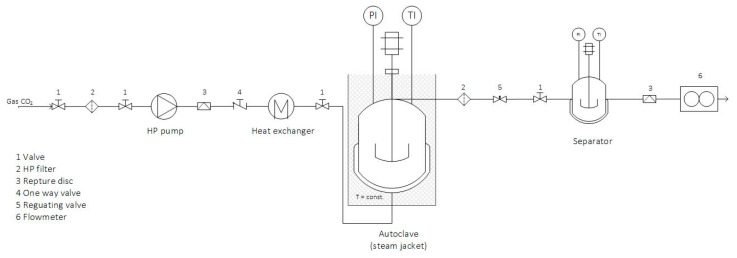
Supercritical fluid extraction system.

The supercritical experiments were performed in an SFE system shown in Figure 7. The ground mixed material (10 g) was placed in an autoclave. The extracts were collected in previously weighed glass tubes. The extraction was performed at different conditions of pressure (200 bar and 300 bar) and temperature (40 °C and 60 °C). The feed of the solvent was F/S = 8.158. The extract was weighed, and the yield was calculated. The extraction experiments with dense gas (CO_2_) were performed on a semi continuous apparatus.

Conventional methods are represented below, abbreviated as SE, UE and CM with the annotation -e, which represents the solvent (ethanol). Supercritical fluid extraction is abbreviated as SFE with the attribution -a, -b, -c and -d, where -a represents 200 bar and 40 °C, -b represents 200 bar and 60 °C, -c represents 300 bar and 40 °C and -d represents 300 bar and 30 °C.

#### 3.2.3. Yield Determination

The yield of the extracts was determined. The extracts were weighed and the yield was gravimetrically calculated as a percentage of the dry weight of the plant. The processes of each extraction method were performed twice. The yield result represents the average value.

#### 3.2.4. Determination of the Extract Effect on the Metabolic Activity of WM-266-4 Cells

Before the extracts were applied to the cancer cells, seven concentrations of the aqueous solutions of the obtained extracts were prepared (c = 50, 20, 10, 5, 1, 0.1, 0.01 mg/mL) with water.

Skin metastatic melanoma cell line WM-266-4 (ATCC^®^ CRL1676™, Manassas, VA, USA) was purchased from American Type Culture Collection (ATCC, Manassas, VA, USA). The cells were grown in a complete medium containing Eagle’s Minimum Essential Medium (EMEM, ATCC^®^ 30-2003™, Kemomed, Kranj, Slovenia) with 1% foetal bovine serum (FBS, ATCC^®^ 30-2021™, Kemomed, Kranj, Slovenia) and 0.02% MycoZap™ Plus-CL (Lonza, Portsmouth, NH, USA) and incubated at 37 °C, 5% CO_2_, ≥90% RH. The cells were plated at a density of 2 × 10^4^ viable cells per well in 96-well culture plates and cultured for 24 h in EMEM (ATCC, Manassas, VA, USA) to allow cell attachment. Five replicates of the experiment were performed. To measure the cells’ metabolic activity, they were exposed to selected concentrations of extracts and cultured for 24 h. Control cells were cultured for the same time and under the same conditions, without extracts. A WST 8 Colorimetric Cell Viability Kit I (PromoKine, PromoCell, Heidelberg, Germany, EU) was used, following the manufacturer’s instructions. Absorbance was measured spectrophotometrically at 570 nm (background absorbance at 630 nm) in pentaplicate for all samples. The percentage of the cells’ metabolic activity (MA) was calculated with the following equation:

(1) $$\mathrm{MA}=\left(\frac{\left(A_{570}-A_{630}\right)\;\mathrm{test}\;\mathrm{sample}\;\mathrm{value}}{\left(A_{570}-A_{630}\right)\;\mathrm{control}\;\mathrm{value}}\right)\times 100$$

where A represents average value of absorbance calculated from pentaplicates. In addition, cell morphology was observed with an inverted microscope (DM16000B, Leica, Morrisville, NC, USA) using a digital camera (DFC365 FX Leica, Buffalo Grove IL, Leica, Morrisville, NC, USA)

#### 3.2.5. HPLC Analysis

Chromatographic analyses were performed using High-performance liquid chromatography (HPLC) (Agilent 1200, Spectralab, Markham, Canada) with a photodiode array detector (DAD). It was used for detection and recorded at UV/Vis 220 nm. Chromatographic separation of cannabinoids was achieved using a Zorbax SB C-18 column (150 mm × 4.6 mm and a particle size of 3.5 μm, Sigma Aldrich, Darmstadt, Germany). The mobile phase was 75:25 (*v*:*v*) methanol/water with 0.1% glacial acetic acid with flow rate 1.0 mL/min. Injection volume of the samples and standards was 10 mL. HPLC analysis was performed for the obtained products after extraction. Cannabinoids were determined: CBD, CBDA, CBG, CBGA, CBC, CBN. Cannabinoid standards were dissolved in methanol at a concentration of 1 mg/mL. The “quality” of the substance was generally expressed by the total percentage of neutral cannabinoids present in the extract. The total percentage of CBD was calculated as the sum of the percentage of CBD plus the percentage of CBDA; this CBDA value was multiplied by a conversion factor that takes into account the difference in molecular weight between the acidic and neutral forms. “Total CBD concentration” was used to report the CBD content of the extract. CBD and CBDA concentrations were summed using molar concentrations. The sum of molar concentrations was calculated as the total CBD concentration in g/100 g (or wt.%), which compensated for the theoretical loss of CO_2_ [61,79,80].

#### 3.2.6. Emulsification Procedure

Emulsification was performed before applying the extracts to the bacteria. The process was designed for mixing the oil extract with a water-based medium. Each extract (x_11_, x_73_, x_37_) was weighed (75 mg) and heated to 40 °C. Emulsifying agent Tween 20 (T20) (100 μL) was added at 25 °C and Mueller-Hinton broth (MH) (900 μL) was homogenized at 40 °C. All three extracts were suspended in MH using a rotar-stator homogenizer (Homogenizer, Polytron Pt1200, Kinematica AG, Luzern, Switzerland). The extract suspension in (MH) was homogenized at 25,000 rpm.

#### 3.2.7. Determination of Antimicrobial Potential

Measurement of antimicrobial potential was performed using the microdilution method. Cation-adjusted Mueller-Hinton broth (MH) and MH supplemented with lysed horse blood and β-NAD (MH-F broth) were used. MH broth meets the requirements of the ISO Technical specification, ISO/TS 16782, 2016 and the quality control criteria published by EUCAST. The prepared emulsions (Section 3.2.6) were applied to *S. aureus* (MRSA) (ATCC 25923, ATCC, Wesel, Germany), *E. coli* (ATCC 25922, ATCC, Wesel, Germany) and *C. albicans* (ATCC 60193, ATCC, Wesel, Germany). Resazurin blue fluorogenic dye (7-hydroxy-10-oxidophenoxazin-10-yum-3-one, sodium), used as a redox indicator, was prepared at 0.04% by dissolving 0.04 g, vortexed and stored at 4 °C. MH (100 μL) was added to each 96 well plate. The prepared emulsion (100 μL) was then added to the first well and mixed. The dilution process was continued as 100 μL from the first column (in which the concentration was *c* = 75 mg/mL) was moved to the second column and mixed. The procedure was repeated until the tenth column, from which 100 μL of the mixture was discarded. Thus, each of the ten columns contained 100 μL. Concentration decreased by half for each column. The concentrations obtained were *c* = 37.5, 18.75, 9.375, 4.688, 2.344, 1.172, 0.586, 0.293, 0.146 and 0.073 mg/mL. The eleventh and twelfth columns were used for sterility control and negative control. The positive control (50 μL MH and 50 μL diluted extract) did not contain extract, as did the other wells, and the negative control (100 μL MH and 10 μL bacteria) did not contain bacteria. Inoculum density of added bacteria was 10^8^ colony-forming unit per milliliter (CFU mL^−1^), and the volume used was 10 μL. The inoculated microplates were incubated for 24 h at 37 °C and bacterial growth was confirmed with the addition of 30 μL of sterile 0.04% resazurin dye solution. The incubation took place for 4 h at 37 °C. A visible jump where remaining bacterial cells remained was recognizable by the resulting colour. In living bacterial cells, the resazurin blue fluorogenic dye changed from dark blue to pink. Columns without discoloration were considered to be the upper minimum value of inhibitory concentration (MIC). All tests were performed in duplicate.

#### 3.2.8. Determination of Antioxidant Activity

Antioxidant activity (A) was determined according to the DPPH method as described in Reference [81]. The experiment was performed in five replicates and the result represents the average value. The antioxidative activity of the sample is given as a percentage of inhibition relative to the reference solution and was calculated using the equation:

(2) $$A\left[\%\right]=\left(\frac{A_{C}^{0}-A_{S}^{15}}{A_{C}^{0}}\right)\times 100$$

where $A_{C}^{0}A_{C}^{0}$ represents absorbance of the reference solution for 0 min and $A_{S}^{15}A_{S}^{15}$ represents absorbance of the solution for 15 min.

#### 3.2.9. Determination of Total Phenols

The total phenolic (TP) contents of plant extracts were determined using Folin-Ciocalteu reagent (Sigma Aldrich, Darmstadt, Germany) as described in Reference [82] with slight correlation. Samples were put into different test tubes and mixed thoroughly with 2.5 mL Folin-Ciocalteu reagent (pre-diluted 10 times with distilled water). After 5 min, 2 mL of sodium carbonate (Na_2_CO_3_) in a concentration of 75 g/L was added and allowed to react for 5 min at 50 °C in a water bath. Absorbance was measured at 765 nm using microplate reader spectrophotometers (Biotek Synergy 2, Biotek, Bad Friedrichshall Germany) [81]. The experiments were done in five replicates and samples were measured three times. The results represent the average value. A standard curve of gallic acid solution was prepared using a similar procedure. The results were expressed as mg GAE/100 g extract sample.

## 4. Conclusions

Three mixtures were prepared (ginger + hemp in a ratio of 1:1, 7:3 and 3:7) as raw material. Seven extractions (UE, SE, CM and SFE at four different conditions) were performed for each mixture. Antioxidants, total phenols and the response of cancer cells to the extract were measured in all 21 extracts. The two most effective extractions (UE and SFE (300 bar, 60 °C)) were selected for further investigation. All six extracts (x_11_(UE-e), x_11_(SFE-d), x_73_(UE-e), x_73_(SFE-d), x_37_(UE-e), x_37_(SFE-d)) were examined for antimicrobial potential. HPLC analysis was performed on the x_11_ extract for all extractions. We confirmed that CBDA predominates in all extracts and is about twice as high as the content of CBD. SCF-d extract and UE extract showed slightly higher CBD content (32%), whilst UE extract had a higher content of CBG content (6%) than other extracts. SCF-d also had a higher content of CBC (5%).

High contents of total phenols and antioxidants were determined in the extracts in which ginger predominated (x_73_): TP = 631.831 mg GAE/100 g material, A = 79%. Antimicrobial analysis showed the highest potential in the extracts in which hemp predominated (x _37_): MIC (*S. aureus*) = 4.69 mg/mL, MIC (*E. coli*) > 37.5 mg/mL and MIC (*C. albicans*) = 4.69 mg/mL. This indicates that for further research, extracts in which ginger and hemp are in the same ratio should be taken into consideration.

Among the microorganisms investigated, the lowest MIC values were determined for *C. albicans*, 4.69 mg/mL. The same MIC value was determined for all extracts. In the gram-positive *S. aureus* organism, MIC values ranged around 9.38 mg/mL. When determining the MIC value for *E. coli*, colour changes were visible at MIC = 37.5 mg/mL. However, given the colour intensity and clear jumps in the other two microorganisms, we assume that it would be good to check for *E. coli* in even slightly higher concentrations of extract.

Furthermore, the effect of the extracts on metastatic cells (WM-266-4) was investigated and we were able to determine visibly the marginal concentration required to inhibit further cancer cell division (c = 1 mg/mL).

The results indicate that amongst the selected extraction methods and conditions, SFE at 300 bar and 60 °C and UE are the most effective, according to measurements of polyphenols, antimicrobial potential, anticancer efficiency and HPLC analyses. In the future, it would be useful to explore them in the mixture.

## Figures and Tables

**Table 1:** Yields (*η* [%]) obtained for extraction experiment.

Sample	*η* [%]						
	Supercritical Fluid Extraction with CO_2_ (SFE)				Soxhlet Extraction (SE) with	Ultrasonic Extraction (UE) with	Cold Maceration (CM) with
	200 bar, 40 °C	200 bar, 60 °C	300 bar, 40 °C	300 bar, 60 °C	Ethanol	Ethanol	Ethanol
x_11_	3.620	4.877	3.930	5.536	4.560	9.120	4.020
x_73_	3.829	3.965	4.608	5.130	4.320	6.030	4.270
x_37_	4.285	5.022	4.797	5.008	4.740	6.130	3.910

**Table 2:** Antioxidant activity values: A [%] and values of total phenols TP [mg GAE/100g material].

Sample		Supercritical Fluid Extraction with CO_2_ (SFE)				Conventional Extractions with Ethanol		
		200 bar, 40 °C	200 bar, 60 °C	300 bar, 40 °C	300 bar, 60 °C	Soxhlet (SE)	Ultrasonic (UE)	Cold Maceration (CM)
x_11_	Antioxidants [%]	54.552	53.978	56.844	56.001	47.134	48.651	36.817
	Total phenols [mg GAE/100 g material]	304.641	409.552	395.484	556.627	360.630	773.008	316.445
x_73_	Antioxidants [%]	68.543	69.892	60.182	79.872	60.519	56.035	47.134
	Total phenols [mg GAE/100 g material]	603.543	366.151	628.676	511.011	609.910	631.831	317.067
x_37_	Antioxidants [%]	39.076	44.7404	38.604	35.536	42.920	38.402	30.007
	Total phenols [mg GAE/100 g material]	515.265	373.056	418.998	442.804	498.784	598.071	253.023

**Table 3:** Minimum inhibitory concentration MIC [mg/mL] of six different ginger-industrial hemp extracts.

Sample	Extraction	*Staphylococcus aureus*	*Escherichia coli*	*Candida albicans*
		MIC [mg/mL]	MIC [mg/mL]	MIC [mg/mL]
x_11_	UE-e	9.38	>37.5	4.69
x_73_	UE-e	9.38	>37.5	4.69
x_37_	UE-e	4.69	>37.5	4.69
x_11_	SFE-d	9.38	>37.5	4.69
x_73_	SFE-d	18.75	>37.5	4.69
x_37_	SFE-d	9.38	>37.5	4.69

Legend: MIC = Minimum inhibitory concentration, SFE = supercritical fluid extraction (d: 300 bar, 60 °C), UE = ultrasonic extraction (e: Solvent is Ethanol.).

## Supplementary Materials

The following are available online.
