# Supplementary material for: The Influence of Hemp Extract in Combination with Ginger on the Metabolic Activity of Metastatic Cells and Microorganisms
Source: Molecules. 2020 Oct 28;25(21):4992. doi: 10.3390/molecules25214992 (PMC7662229; doi:10.3390/molecules25214992)
Supplement: Supplementary file 1 [file molecules-25-04992-s001.pdf]

Supplementary Materials

The Influence of Hemp Extract in Combination with Ginger on the Metabolic Activity of Metastatic Cells and Microorganisms

Taja Žitek [1], Maja Leitgeb [1,2], Andrej Golle [3], Barbara Dariš [2], Željko Knez [1,2] and Maša Knez Hrnčič [1,*]

[1] Laboratory for Separation Processes and Product Design, Faculty of Chemistry and Chemical Engineering, University of Maribor, SI-2000 Maribor, Slovenia; taja.zitek@um.si (T.Z.); maja.leitgeb@um.si (M.L.); zeljko.knez@um.si (Z.K.)

[2] Faculty of Medicine, University of Maribor, Taborska 8, SI-2000 Maribor, Slovenia; barbara.daris@um.si

[3] National Laboratory for Health, Environment and Food, Prvomajska ulica 1, SI-2000 Maribor, Slovenia; andrej.golle@nlzoh.si

* Correspondence: masa.knez@um.si; Tel.:+00386-2-229-44-70

Academic Editor: Celestino Santos-Buelga

Received: 02 October 2020; Accepted: 26 October 2020; Published: 27 October 2020

Table S1: Cannabinoid composition of *Cannabis Sativa L.* (KC Dora) used in experiments. Analysis were done by duplicate and values expressed as cannabinoid percentage as mean ± SD.

Cannabinoids:	**SCF-a**	**SCF-b**	**SCF-c**	**SCF-d**	**SE-e**	**UE-e**	**CM-e**
**CBD %**	19.50 ± 0.02	26.63 ± 0.04	22.13 ± 0.02	30.51 ± 0.02	20.63 ± 0.13	33.51± 0.53	21.03 ± 0.57
**CBDA %**	53.25 ± 0.98	45.75 ± 1.01	53.26 ± 1.40	45.01 ± 0.05	49.75 ± 0.82	45.06 ± 0.72	52.76 ± 0.92
**CBC %**	1.43 ± 0.02	1.55 ± 0.11	1.46 ± 0.01	5.48 ± 0.13	4.51 ± 0.02	1.64 ± 0.02	1.65 ± 0.01
**CBN %**	N.D.	N.D.	0.06 ± 0.02	0.38 ± 0.19	0.20 ± 0.14	N.D.	N.D.
**CBGA %**	1.41 ± 0.09	0.38 ± 0.13	1.76 ± 0.08	0.86 ± 0.14	4.42 ± 0.19	1.51 ± 0.17	6.04 ± 0.18
**CBG %**	1.52 ± 0.01	1.88 ± 0.07	2.06 ± 0.09	3.38 ± 0.01	2.23 ± 0.26	5.75 ± 0.18	1.12 ± 0.14

N.D. = no detectable

SFE= supercritical fluid extraction (a: 200 bar, 40°C, b: 200 bar, 60°C, c: 300 bar, 40°C d: 300 bar, 60°C), SE = Soxhlet extraction, UE = ultrasonic extraction, CM = cold maceration (e: solvent is ethanol).
